# Supplementary material for: Phylogenomics uncovers early hybridization and adaptive loci shaping the radiation of Lake Tanganyika cichlid fishes
Source: Nat Commun. 2018 Aug 8;9:3159. doi: 10.1038/s41467-018-05479-9 (PMC6082878; doi:10.1038/s41467-018-05479-9)
Supplement: Supplementary file 1 — Supplementary Information [file 41467_2018_5479_MOESM1_ESM.pdf]

Supplementary Information

Phylogenomics uncovers early hybridization and adaptive loci shaping the radiation of Lake Tanganyika cichlid fishes

Irisarri et al.

Supplementary Table 1. Details of taxon sampling. For each individual, the species name, tribe and ID in alignment files is shown.

ID	Species	Tribe	ID	Species	Tribe
I9125_1_36	*Bathybates fasciatus*	Bathybatini	I9130_1_41	*Julidochromis marlieri*	Lamprologini
I9151_1_62	*Bathybates fasciatus*	Bathybatini	I9142_1_53	*Julidochromis ornatus*	Lamprologini
I9137_1_48	*Bathybates graueri*	Bathybatini	I9154_1_65	*Julidochromis regani*	Lamprologini
I9161_1_72	*Bathybates minor*	Bathybatini	I9166_1_77	*Lamprologus callipterus*	Lamprologini
I9203_2_20	*Hemibates stenosoma*	Bathybatini	I9178_1_89	*Lamprologus lemairii*	Lamprologini
I9215_2_32	*Hemibates stenosoma*	Bathybatini	I9096_1_7	*Lamprologus ocellatus*	Lamprologini
I9102_1_13	*Boulengerochromis microlepis*	Boulengerochromini	I9107_1_18	*Lamprologus ornatipinnis*	Lamprologini
I9114_1_25	*Boulengerochromis microlepis*	Boulengerochromini	I9131_1_42	*Lepidiolamprologus boulengeri*	Lamprologini
I9174_1_85	*Chromidotilapia guntheri*	Chromidotilapiini	I9143_1_54	*Lepidiolamprologus elongatus*	Lamprologini
I9159_1_70	*Pelvicachromis pulcher*	Chromidotilapiini	I9155_1_66	*Lepidiolamprologus meeli*	Lamprologini
Ac_T88_LOC	*Andinoacara coeruleopunctatus*	Cichlasomatini	I9167_1_78	*Lepidiolamprologus nkambae*	Lamprologini
I9257_2_74	*Coptodon nyongana*	Coptodonini	Nb_T88_LOC	*Neolamprologous brichardi*	Lamprologini
I9092_1_3	*Ctenochromis benthicola*	Cyphotilapiini	I9156_1_67	*Neolamprologus brevis*	Lamprologini
I9103_1_14	*Ctenochromis benthicola*	Cyphotilapiini	I9168_1_79	*Neolamprologus buescheri*	Lamprologini
I9163_1_74	*Cyphotilapia frontosa*	Cyphotilapiini	I9180_1_91	*Neolamprologus caudopunctatus*	Lamprologini
I9175_1_86	*Cyprichromis coloratus*	Cyprichromini	I9098_1_9	*Neolamprologus christyi*	Lamprologini
I9093_1_4	*Cyprichromis leptosoma*	Cyprichromini	I9109_1_20	*Neolamprologus cylindricus*	Lamprologini
I9104_1_15	*Cyprichromis microlepidotus*	Cyprichromini	I9133_1_44	*Neolamprologus falcicula*	Lamprologini
I9123_1_34	*Paracyprichromis brieni*	Cyprichromini	I9145_1_56	*Neolamprologus fasciatus*	Lamprologini
I9172_1_83	*Asprotilapia leptura*	Ectodini	I9157_1_68	*Neolamprologus furcifer*	Lamprologini
I9113_1_24	*Aulonocranus dewindtii*	Ectodini	I9169_1_80	*Neolamprologus helianthus*	Lamprologini
I9126_1_37	*Callochromis macrops*	Ectodini	I9181_1_92	*Neolamprologus leleupi*	Lamprologini
I9138_1_49	*Callochromis pleurospilus*	Ectodini	I9099_1_10	*Neolamprologus modestus*	Lamprologini
I9150_1_61	*Cardiopharynx schoutedeni*	Ectodini	I9110_1_21	*Neolamprologus multifasciatus*	Lamprologini
I9127_1_38	*Cunningtonia longiventralis*	Ectodini	I9122_1_33	*Neolamprologus savoryi*	Lamprologini
I9139_1_50	*Cyathopharynx furcifer*	Ectodini	I9134_1_45	*Neolamprologus sexfasciatus*	Lamprologini
I9116_1_27	*Ectodus descampsii*	Ectodini	I9146_1_57	*Neolamprologus tetracanthus*	Lamprologini
I9128_1_39	*Enantiopus melanogenys*	Ectodini	I9158_1_69	*Neolamprologus tretocephalus*	Lamprologini
I9105_1_16	*Grammatotria lemairii*	Ectodini	I9100_1_11	*Palaeolamprologus toae*	Lamprologini
I9179_1_90	*Lestradea perspicax*	Ectodini	I9209_2_26	*Telmatochromis dhonti*	Lamprologini
I9132_1_43	*Microdontochromis rotundiventralis*	Ectodini	I9221_2_38	*Telmatochromis temporalis*	Lamprologini
I9144_1_55	*Microdontochromis tenuidentatus*	Ectodini	I9233_2_50	*Telmatochromis vittatus*	Lamprologini
I9238_2_55	*Xenotilapia caudafasciata*	Ectodini	I9214_2_31	*Variabilichromis moorii*	Lamprologini
I9250_2_67	*Xenotilapia flavipinnis*	Ectodini	I9173_1_84	*Benthochromis horii*	Limnochromini
I9262_2_79	*Xenotilapia spiloptera*	Ectodini	I9091_1_2	*Benthochromis melanoides*	Limnochromini
I9208_2_25	*Eretmodus cyanostictus*	Eretmodini	I9176_1_87	*Gnathochromis permaxillaris*	Limnochromini
I9216_2_33	*Eretmodus marksmithi*	Eretmodini	I9117_1_28	*Greenwoodochromis christyi*	Limnochromini

Supplementary Table 1 (Cont.)

I9268_2_85	*Spathodus erytrhodon*	Eretmodini	I9097_1_8	*Limnochromis auritus*	Limnochromini
I9240_2_57	*Spathodus marlieri*	Eretmodini	I9196_2_13	*Reganochromis calliurus*	Limnochromini
I9197_2_14	*Tanganicodus irsacae*	Eretmodini	I9187_2_4	*Triglachromis otostigma*	Limnochromini
I9228_2_45	*Tanganicodus irsacae*	Eretmodini	I9182_1_93	*Oreochromis tanganicae*	Oreochromini
I9152_1_63	*Etroplus maculatus*	Etroplinae	I9220_2_37	*Sarotherodon galilaeus*	Oreochromini
I9164_1_75	*Etroplus suratensis*	Etroplinae	On_T88_LOC	*Oreochromis niloticus*	Oreochromnini
I9135_1_46	*Paretroplus menarambo*	Etroplinae	I9274_2_91	*Orthochromis uvinzae*	Orthochromini
I11182_14833	*Steatocranus irvinei*	Gobiocichlinae	I9147_1_58	*Pelmatochromis buettikoferi*	Pelmatochromini
I9090_1_1	*Astatoreochromis alluaudi*	Haplochromini	I9141_1_52	*Haplotaxodon microlepis*	Perissodini
I9101_1_12	*Astatoreochromis alluaudi*	Haplochromini	I9153_1_64	*Haplotaxodon trifasciatus*	Perissodini
Ab_T88_LOC	*Astatotilapia burtoni*	Haplochromini	I9171_1_82	*Perissodus microlepis*	Perissodini
I9265_2_82	*Astatotilapia burtoni*	Haplochromini	I9231_2_48	*Plecodus sp.*	Perissodini
I9229_2_46	*Astatotilapia calliptera*	Haplochromini	I9226_2_43	*Xenochromis hecqui*	Perissodini
I9194_2_11	*Astatotilapia stappersi*	Haplochromini	I11183_14834	*Paratilapia polleni*	Ptychochrominae
I9264_2_81	*Copadichromis borleyi*	Haplochromini	I9267_2_84	*Ptychochromis grandidieri*	Ptychochrominae
I9205_2_22	*Copadichromis mloto*	Haplochromini	I9185_2_2	*Ptychochromis oligacanthus*	Ptychochrominae
I9252_2_69	*Cynotilapia afra*	Haplochromini	I9186_2_3	*Steatocranus casuarius*	Steatocranini
I9129_1_40	*Haplochromis nubilus*	Haplochromini	I11181_14624	*Tilapia sparrmanii*	Tilapiini
I9253_2_70	*Haplochromis nyereri*	Haplochromini	I9115_1_26	*Ctenochromis horei*	Tropheini
I9217_2_34	*Lethrinops marginatus*	Haplochromini	I9094_1_5	*Gnathochromis pfefferi*	Tropheini
I9241_2_58	*Melanochromis auratus*	Haplochromini	I9106_1_17	*Interochromis loocki*	Tropheini
Mz_T88_LOC	*Metriaclima Zebra Brawand*	Haplochromini	I9108_1_19	*Limnotilapia dardennii*	Tropheini
I9243_2_60	*Pseudocrenilabrus nicholsi*	Haplochromini	I9120_1_31	*Lobochilotes labiatus*	Tropheini
I9239_2_56	*Pseudocrenilabrus philander*	Haplochromini	I9204_2_21	*Petrochromis famula*	Tropheini
Pn_T88_LOC	*Pundamilia nyererei*	Haplochromini	I9234_2_51	*Petrochromis famula*	Tropheini
I9165_1_76	*Hemichromis cerasogaster*	Hemichromini	I9218_2_35	*Petrochromis fasciolatus*	Tropheini
I9177_1_88	*Hemichromis sp.*	Hemichromini	I9254_2_71	*Petrochromis polyodon*	Tropheini
I9136_1_47	*Amphilophus astorquii*	Heroini	Pt_T88_LOC	*Petrochromis Trewavasae*	Tropheini
Midas_T88	*Amphilophus cintrinellus*	Heroini	I9255_2_72	*Pseudosimochromis curvifrons*	Tropheini
I9148_1_59	*Amphilophus citrinellus*	Heroini	I9244_2_61	*Simochromis diagramma*	Tropheini
I9160_1_71	*Amphilophus zaliosus*	Heroini	I11194_14847	*Tropheus annectens*	Tropheini
I9111_1_22	*Parachromis managuense*	Heroini	I11192_14845	*Tropheus brichardi*	Tropheini
I9095_1_6	*Heterochromis multidens*	Heterochromidini	I9119_1_30	*Tropheus duboisi*	Tropheini
I11187_14840	*Heterotilapia buttikoferi*	Heterotilapiini	Tm_T88_LOC	*Tropheus moorii*	Tropheini
I9112_1_23	*Altolamprologus calvus*	Lamprologini	I9191_2_8	*Tylochromis lateralis*	Tylochromini
I9124_1_35	*Altolamprologus compressiceps*	Lamprologini	I9227_2_44	*Tylochromis polylepis*	Tylochromini
I9162_1_73	*Chalinochromis bifrenatus*	Lamprologini	I9202_2_19	*Tylochromis sudanensis*	Tylochromini
I9118_1_29	*Julidochromis dickfeldii*	Lamprologini			

Supplementary Table 2. Calibration schemes C01-C10. Each scheme includes several vicariant (VIC) or fossil (FOS) calibrations. Minimum and maximum ages are in million years ago (Ma) and in all cases relevant references are indicated. Coloured cells indicate that the calibration is included into the corresponding scheme, shown as blue (for vicariance-only), brown (for fossil-only), or green (fossil-only but including upper bounds for vicariance nodes) for schemes C01-C05. Schemes C06-C10 are shown in orange, and correspond to C01-05 respectively but additionally include the new fossil *T. pickfordi*.

C01	C02	C03	C04	C05	C06	C07	C08	C09	C10	Calibration	Min.	Max.	Calibrated nodes	References
blue					orange					VIC1	121	165	Madagascar+India/. Africa+South America	Gondwana fragmentation[1], consistent with Genner et al.[2]
blue					orange					VIC2	86	101	Africa/ South America	Gondwana fragmentation[1], consistent with Genner et al.[2]
blue					orange					VIC3	63	88	Madagascar / India	Gondwana fragmentation[1], consistent with Genner et al.[2]
			green	green				orange	orange	VIC1-MAX	–	165	Madagascar+India/. Africa+South America	Gondwana fragmentation[1], consistent with Genner et al.[2]
			green	green				orange	orange	VIC2-MAX	–	101	Africa/ South America	Gondwana fragmentation[1], consistent with Genner et al.[2]
			green	green				orange	orange	VIC3-MAX	–	88	Madagascar / India	Gondwana fragmentation[1], consistent with Genner et al.[2]
	brown	brown	green	green		orange	orange	orange	orange	FOS1	40	101	Cichlasomatini / Heroini	Min. based on Neotropical cichlids †*Plesioheros* and †*Tremembichthys* from Eocene deposits[3–5]. Conservative max. as max. age of continental split Africa / S. America.
	brown		green			orange		orange		FOS2	45.7	101	First split within African cichlids	Min. based on the African †*Mahengechromis* sp. from Eocene[6–8]. Conservative upper bound as max. age of continental split Africa / S. America.
		brown		green			orange		orange	FOS2b	45.7	101	Second split within African cichlids (excl. *Heterochromis*)	Min. based on the African †*Mahengechromis* sp. from Eocene[6–8]. Conservative upper bound as max. age of continental split Africa / S. America.
	brown	brown	green	green		orange	orange	orange	orange	FOS3	9.3	62	Haplotilapiines (without Etiini, not included in our data)	Min. based on the oldest Oreochromini †*Sarotherodon martini* from Lake Turkana[9,10]. Conservative max. bound corresponding to upper 95% CI from Genner et al.[2]
	brown	brown	green	green		orange	orange	orange	orange	FOS4	5.98	47.5	Oreochromini	Min. based on the oldest *Oreochromis* †*O. lorenzoi* from the Late Miocene[11]. Conservative max. bound corresponding to upper 95% CI from Genner et al.[2]
	brown	brown	green	green		orange	orange	orange	orange	FOS5	33.1	79.6	African cichlids (excl. *Heterochromis*)	Min. based on a cf. *Tylochromis* fossil from Late Eocene–Early Oligocene deposits in Egypt[12]. Conservative max. bound corresponding to upper 95% CI from Genner et al.[2]
					orange	orange	orange	orange	orange	FOS6	9.3	43.2	H-lineage / Lamprologini	Min. based on *Tugenchromis pickfordi*[13]. Conservative max. bound corresponding to upper 95% CI from Genner et al.[2]

Supplementary Table 3. Summary of Patterson's *D* tests. For each hypotheses tested, the following information is shown: test groups and outgroup, average numbers of informative, ABBA and BABA sites and the distribution of *D*-statistics (violin plots). $D < 0$ indicates gene flow between P1 and P3 and $D > 0$ indicates gene flow between P2 and P3. LT, LV and LM refer respectively to species flocks of Lake Tanganyika, Lake Victoria and Lake Malawi. All tests except H2d1 and H2d2 were significant (Z-score > 3, Benjamini-Hochberg-adjusted $p < 0.05$). Full details details of each permutation can be found in Supplementary Data 2.

**Hypothesis**	**Test groups<br>P1, P2, P3, O<br>[No. individuals]**	**Average informative sites**	**Averag e ABBA sites**	**Averag e BABA sites**	**Distribution of *D*-statistics**
H1a1	P1: LT 'modern' tribes [111]<br>P2: Boulengerochromini [2]<br>P3: Steatocranini [1]<br>O: *Tilapia sparmanii*	23,917	227	1,070	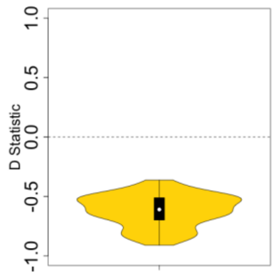
H1a2	P1: LT 'modern' tribes [111]<br>P2: Boulengerochromini [2]<br>P3: Steatocranini [1]<br>O: *Heterotilapia buttikoferi*	23,223	204	1,139	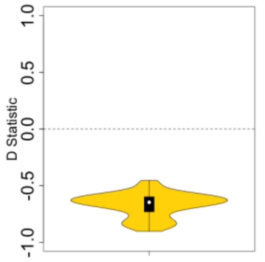
H1a3	P1: LT 'modern' tribes [111]<br>P2: Boulengerochromini [2]<br>P3: Steatocranini [1]<br>O: *Pelmatochromis buettikoferi*	43,999	475	1,101	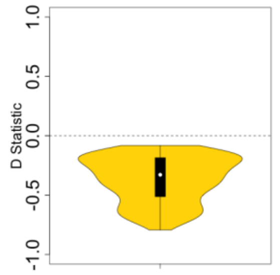
H1b1	P1: LT 'modern' tribes [111]<br>P2: Bathybatini [6]<br>P3: Steatocranini [1]<br>O: *Tilapia sparmanii*	27,291	244	1,126	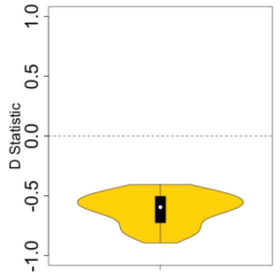
H1b2	P1: LT 'modern' tribes [111]<br>P2: Bathybatini [6]<br>P3: Steatocranini [1]<br>O: *Heterotilapia buttikoferi*	26,828	248	1166	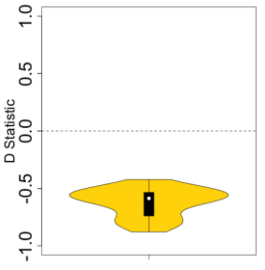

H1b3	P1: LT 'modern' tribes [111]<br>P2: Bathybatini [6]<br>P3: Steatocranini [1]<br>O: *Pelmatochromis buettikoferi*	47,600	525	1,255	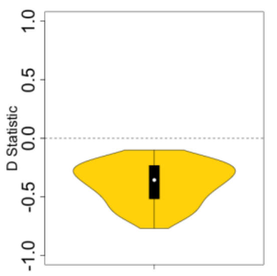
H1c1	P1: LT 'modern' tribes [111]<br>P2: Boulengerochromini [2]<br>P3: Heterotilapiini [1]<br>O: *Pelmatochromis buettikoferi*	42,718	554	286	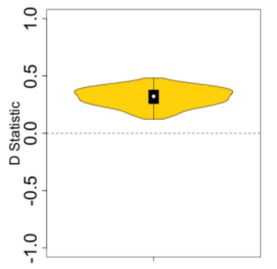
H1c2	P1: LT 'modern' tribes [111]<br>P2: Bathybatini [6]<br>P3: Heterotilapiini [1]<br>O: *Pelmatochromis buettikoferi*	46,583	632	444	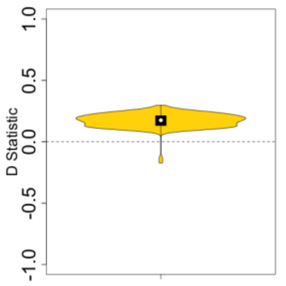
H1c3	P1: LT 'modern' tribes [111]<br>P2: Boulengerochromini [2]<br>P3: Hemichromini [2]<br>O: *Pelmatochromis buettikoferi*	53,309	322	435	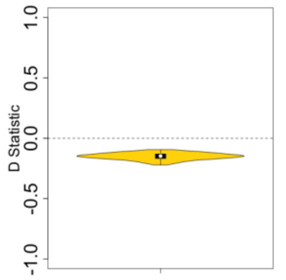
H1c4	P1: LT 'modern' tribes [111]<br>P2: Bathybatini [6]<br>P3: Hemichromini [2]<br>O: *Pelmatochromis buettikoferi*	62,236	470	511	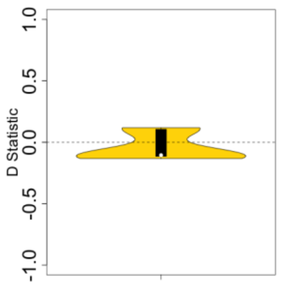
H2a1	P1: Tropheini [14]<br>P2: Orthochromini [1]<br>P3: Cyphotilpiini [3]<br>O: *Tilapia sparmanii*	21,444	312	448	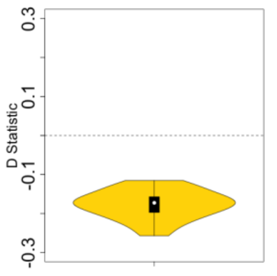

H2a2	P1: Tropheini [14]<br>P2: Orthochromini [1]<br>P3: Cyphotilpiini [3]<br>O: *Heterotilapia buttikoferi*	21,044	302	420	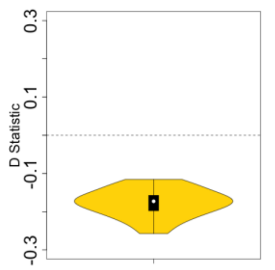
H2b1	P1: LM, LV [24]<br>P2: Orthochromini [1]<br>P3: Cyphotilpiini [3]<br>O: *Tilapia sparmanii*	22,205	320	429	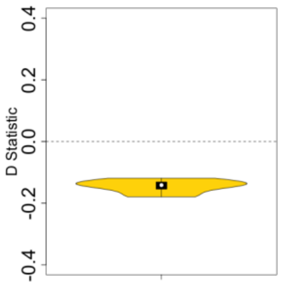
H2b2	P1: LM, LV [24]<br>P2: Orthochromini [1]<br>P3: Cyphotilpiini [3]<br>O: *Heterotilapia buttikoferi*	21,713	304	407	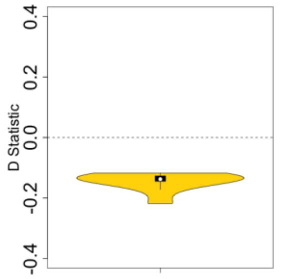
H2c1	P1: *Astatoreochromis* [2]<br>P2: Orthochromini [1]<br>P3: Cyphotilpiini [3]<br>O: *Tilapia sparmanii*	21,790	296	422	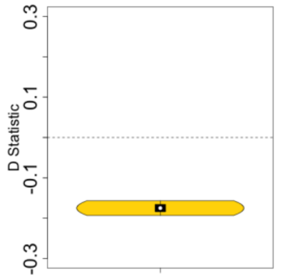
H2c2	P1: *Astatoreochromis* [2]<br>P2: Orthochromini [1]<br>P3: Cyphotilpiini [3]<br>O: *Heterotilapia buttikoferi*	21,217	289	412	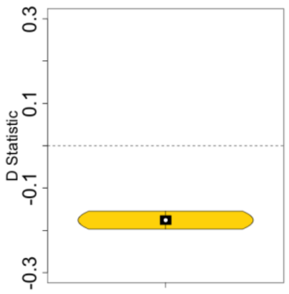
H2d1	P1: *Pseudocrenilabrus* [2]<br>P2: Orthochromini [1]<br>P3: Cyphotilpiini [3]<br>O: *Tilapia sparmanii*	24,118	371	377	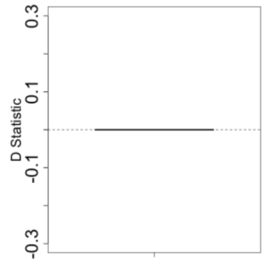

H2d2	P1:*Pseudocrenilabrus* [2]<br>P2: Orthochromini [1]<br>P3: Cyphotilpiini [3]<br>O: *Heterotilapia buttikoferi*	23,864	339	365	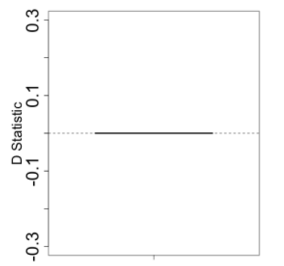
H3	P1: Perissodini [5]<br>P2: Cyprichromini [3]<br>P3: Benthochromini [2]<br>O: *Tilapia sparmanii*	19,057	318	318	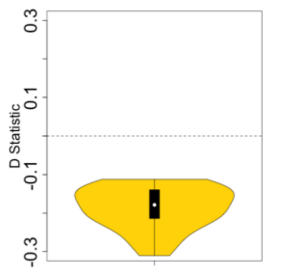
H4a	P1: Lamprologini [36]<br>P2: H-lineage [52]<br>P3: Boulengerochromini [2]<br>O: *Tilapia sparmanii*	24,059	337	264	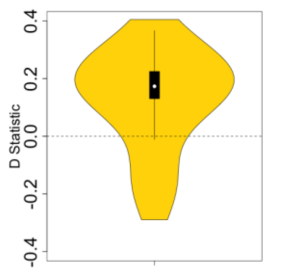
H4b	P1: Lamprologini [36]<br>P2: H-lineage [52]<br>P3: Bathybatini [6]<br>O: *Tilapia sparmanii*	27,480	300	398	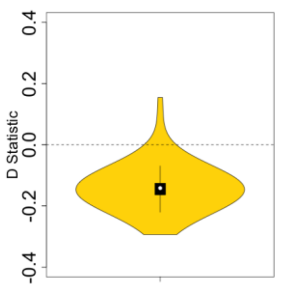
H5a	P1: Lamprologini [36]<br>P2: Perissodini+ Cyprichromini [8]<br>P3: Boulengerochromini [2]<br>O: *Tilapia sparmanii*	23,263	340	224	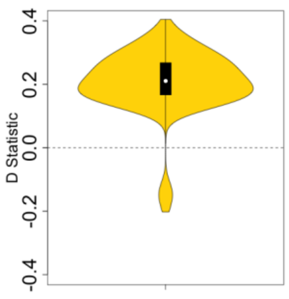
H5b	P1: Lamprologini [36]<br>P2: Perissodini+ Cyprichromini [8]<br>P3: Bathybatini [6]<br>O: *Tilapia sparmanii*	26,968	287	382	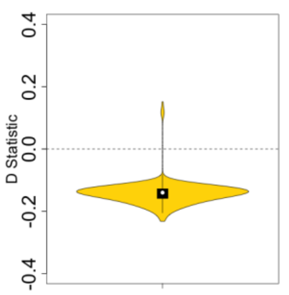

Supplementary Table 4. Summary of gene ontology (GO) annotations for loci with stronger hybridization signal between Steatocranini and the Lake Tanganyika 'modern' tribes (Fig. 3b).

GO term	Description	Freq.	GO term	Description	Freq.
GO:0006810	transport	17,62%	GO:0006334	nucleosome assembly	0,09%
GO:0055114	oxidation-reduction process	15,06%	GO:0007517	muscle organ development	0,08%
GO:0050896	response to stimulus	12,21%	GO:0048511	rhythmic process	0,08%
GO:0006351	transcription, DNA-templated	10,66%	GO:0006529	asparagine biosynthetic process	0,07%
GO:0006355	regulation of transcription, DNA-templated	9,92%	GO:0003007	heart morphogenesis	0,06%
GO:0055085	transmembrane transport	8,92%	GO:0031023	microtubule organizing center organization	0,06%
GO:0016310	phosphorylation	7,76%	GO:0043570	maintenance of DNA repeat elements	0,06%
GO:0007154	cell communication	7,22%	GO:0007219	Notch signaling pathway	0,06%
GO:0007165	signal transduction	6,62%	GO:0007623	circadian rhythm	0,06%
GO:0005975	carbohydrate metabolic process	5,26%	GO:0007131	reciprocal meiotic recombination	0,05%
GO:0006508	proteolysis	5,22%	GO:0007601	visual perception	0,05%
GO:0006468	protein phosphorylation	4,14%	GO:0009411	response to UV	0,05%
GO:0035556	intracellular signal transduction	4,00%	GO:0051216	cartilage development	0,04%
GO:0006629	lipid metabolic process	3,52%	GO:0060041	retina development in camera-type eye	0,04%
GO:0006812	cation transport	3,24%	GO:0007020	microtubule nucleation	0,04%
GO:0006396	RNA processing	3,21%	GO:0032012	regulation of ARF protein signal transduction	0,04%
GO:0032259	methylation	3,10%	GO:0051103	DNA ligation involved in DNA repair	0,04%
GO:0008652	cellular amino acid biosynthetic process	2,93%	GO:0008593	regulation of Notch signaling pathway	0,03%
GO:0000160	phosphorelay signal transduction system	2,57%	GO:0045910	negative regulation of DNA recombination	0,03%
GO:0006281	DNA repair	2,23%	GO:0006829	zinc II ion transport	0,03%
GO:0007049	cell cycle	1,89%	GO:0071577	zinc II ion transmembrane transport	0,03%
GO:0030001	metal ion transport	1,68%	GO:0048747	muscle fiber development	0,02%
GO:0006310	DNA recombination	1,64%	GO:0060042	retina morphogenesis in camera-type eye	0,02%
GO:0007275	multicellular organism development	1,56%	GO:0007004	telomere maintenance via telomerase	0,02%
GO:0046907	intracellular transport	1,56%	GO:0001756	somitogenesis	0,02%
GO:0051301	cell division	1,23%	GO:0008630	intrinsic apoptotic signaling pathway in response to DNA damage	0,02%
GO:0006644	phospholipid metabolic process	1,21%	GO:0046579	positive regulation of Ras protein signal transduction	0,02%
GO:0006886	intracellular protein transport	1,20%	GO:0035195	gene silencing by miRNA	0,02%
GO:0016192	vesicle-mediated transport	1,09%	GO:0007602	phototransduction	0,02%
GO:0016051	carbohydrate biosynthetic process	1,08%	GO:0051289	protein homotetramerization	0,02%
GO:0006457	protein folding	0,90%	GO:0000042	protein targeting to Golgi	0,02%
GO:0007186	G-protein coupled receptor signaling pathway	0,88%	GO:0008045	motor neuron axon guidance	0,01%
GO:0007010	cytoskeleton organization	0,79%	GO:0045190	isotype switching	0,01%
GO:0006470	protein dephosphorylation	0,59%	GO:0000710	meiotic mismatch repair	0,01%
GO:0016567	protein ubiquitination	0,52%	GO:0048741	skeletal muscle fiber development	0,01%
GO:0007264	small GTPase mediated signal transduction	0,49%	GO:0043113	receptor clustering	0,01%
GO:0006541	glutamine metabolic process	0,47%	GO:0060037	pharyngeal system development	0,01%
GO:0016042	lipid catabolic process	0,40%	GO:0033333	fin development	0,01%
GO:0007067	mitotic nuclear division	0,32%	GO:0034453	microtubule anchoring	0,01%
GO:0006486	protein glycosylation	0,32%	GO:0032402	melanosome transport	0,01%
GO:0000910	cytokinesis	0,32%	GO:0002053	positive regulation of mesenchymal cell proliferation	0,01%
GO:0007018	microtubule-based movement	0,29%	GO:0010043	response to zinc ion	0,01%
GO:0006298	mismatch repair	0,17%	GO:0033292	T-tubule organization	0,00%
GO:0008154	actin polymerization or depolymerization	0,15%	GO:0016203	muscle attachment	0,00%
GO:0006614	SRP-dependent cotranslational protein targeting to membrane	0,14%	GO:0060536	cartilage morphogenesis	0,00%
GO:0009103	lipopolysaccharide biosynthetic process	0,14%	GO:0033334	fin morphogenesis	0,00%
GO:0007507	heart development	0,13%	GO:0033339	pectoral fin development	0,00%
GO:0035023	regulation of Rho protein signal transduction	0,13%	GO:0060876	semicircular canal formation	0,00%
GO:0006888	ER to Golgi vesicle-mediated transport	0,13%	GO:0016446	somatic hypermutation of immunoglobulin genes	0,00%
GO:0016032	viral process	0,13%	GO:0070121	Kupffer's vesicle development	0,00%
GO:0043401	steroid hormone mediated signaling pathway	0,10%	GO:0030206	chondroitin sulfate biosynthetic process	0,00%
GO:0018298	protein-chromophore linkage	0,10%	GO:0051322	anaphase	0,00%
GO:0007156	homophilic cell adhesion via plasma membrane adhesion molecules	0,10%			

Supplementary Table 5. Genes associated with key innovations in cichlids analysed in this study. Genes are classified as associated with colour vision, coloration, or tooth and bone (jaw) development; known functions and relevant references are shown.

**Colour vision**	
lws	Long wave (red) sensitive opsin[14]
sws1	Short wave (ultraviolet) sensitive opsin[14]
sws2a	Short wave (blue) sensitive opsin[14]
sws2b	Short wave (violet) sensitive opsin[14]
rh2b	Short wave (blue-green) sensitive opsin[14]
rh2aα	Mid wave (green) sensitive opsin[14]
rh2aβ	Mid wave (green) sensitive opsin[14]
**Coloration**	
csf1ra	Under positive selection in halochromines, xantophores, eggspots[15]
dlc	Stripe patterning in zebrafish, melanophore-xantophore interaction[16]
fbxo36b	Positional candidate for stripe patterning in *Haplochromis sauvagei*[17]
hag	Stripe patterning in zebrafish[18], increased splicing diversity[19] and positive selection[20] in haplochromines
kir7.1	Stripe patterning in zebrafish[21,22]
kir7.2	Cichlid-specific duplicate, evidence for neofunctionalization[23]
kita	Melanophore development and proliferation in zebrafish[24]
kitla	Melanophore development and proliferation in stickleback and human[25]
mitfa	Melanophore survival and viability in mice[26] and human disease models[27,28]
smtlb	Proliferation and morphogenesis of pigment cells and mating preference in medaka[29–31]
sox10	Pigment cell proliferation and viability in zebrafish[32]
**Tooth and bone development**	
barx1	Involved in dental regulatory circuit; expressed in cichlid lower pharyngeal jaw[33]
bmp2	Involved in odontogenesis in mice[33]; upregulated in hard diet in Gunter et al.[34]; osteoblast proliferation and differentiation pathway; early neurocrest inducer[35]
bmp4	Involved in odontogenesis in mice[33]; craniofacial development[36]; early neurocrest inducer[35]
col6a1	Collagen gene, important in bone development; expressed in cichlid lower pharyngeal jaw
creb1	Transcription factor with binding sites in gene network of Schneider et al.[37]
c-fos	Transcription factor with binding sites in gene network of Schneider et al.[37]
dlx2	Involved in dental regulatory circuit; expressed in cichlid lower pharyngeal jaw[33]
pitx2	Involved in dental regulatory circuit; expressed in cichlid lower pharyngeal jaw[33]
runx2b	Involved in odontogenesis in mice[33]; upregulated in hard diet in Gunther et al.[34]; osteoblast proliferation and differentiation pathway
ssh	Involved in dental regulatory circuit, expressed in cichlid lower pharyngeal jaw[33]
sp7	(=*osx*) Gene of the extracellular matrix and involved in osteoblast proliferation and differentiation; upregulated in hard diet in Gunter et al.[34]

Supplementary Table 6. Anchored loci not associated with a particular cichlid innovation but displaying a dN/dS>0.4 (Fig. 4a). For each locus, the dN/dS, annotation (as *Oreochromis niloticus* gene ID and Ensembl ID), and potential functions are shown.

Locus	dN/dS	*O. niloticus gene ID*	Ensembl ID	Putative function
L49	0.56586	*on.gene.LG13.372*	ENSGACG00000005370 GOLGA4	Golgi aparatus
L411	0.55249	*on.gene.LG14.742*	ENSDARG00000002336 dlc	ligand for notch receptors, notch pathway key to chondrogenesis and mice mandible. Involved in somitogenesis.
L23	0.50997	*on.gene.LG15.706*	ENSGACG00000004820 TDRD6	reproductive system. This gene encodes a tudor domain-containing protein and component of the chromatoid body, a type of ribonucleoprotein granule present in male germ cells. Studies in rodents have demonstrated a role in spermiogenesis and the nonsense mediated decay (NMD) pathway
L193	0.46690	*N/A*	P. nyeyerei glucosaminyl (N-acetyl) transferase 3, mucin type (gcnt3) (LOC101474413)	digestive system and immune system
L99	0.45597	*N/A*	P. nyeyerei AB A-kinase anchor protein 12-like (LOC102210822)	growth and craniofacial development in mice, muscle protein
L217	0.44572t	*on.gene.LG9.335*	ENSGACG00000004197 EMILIN2 elastin microfibril interfacter 2	development and morphogenesis

Supplementary Table 7. Analysis of coefficients of selection (ω or dN/dS) on genes associated with key innovations. Table shows general properties of alignments, coefficient of selection under random sites M0 model and two tests of positive selection (M3/M0 and M2a/M1a) estimated with PAML. Significance levels were corrected using the Benjamini-Hochberg correction for multiple testing.

						Likelihood ratio tests		Parameters under M2a[c]		Positively selected sites (M2a, BEB)[d]
**Gene**	**s[a]**	**p_{US}[a]**	**ls[a]**	**p_{CDS}[a]**	**ω_{M0}**	**M3/M0**	**M2a/M1a**	**ω_0 (p_0)**	**ω_2 (p_2)**	
Genes involved in coloration										
csf1ra	146	(1.0)	979	(1.0)	0.220	339.80*	19.38*	0.094 (0.83)	3.66 (0.002)	99, 100
dlc	144	(1.0)	722	(1.0)	0.197	1228*	89.34*	0.087 (0.80)	4.24 (0.03)	**94**, 114, 403, **489**, 704, 708, 709, 714, 720, 721
fbxo36b	134	(0.7)	153	(0.5)	0.463	16.49*	0.15^{NS}	0.199 (0.68)	1.13 (0.31)	
hag	141	(0.9)	389	(1.0)	0.203	284.82*	0.13^{NS}	0.061 (0.84)	0 (0)	
kir7.1	145	(0.8)	363	(1.0)	0.146	63.22*	0^{NS}	0.075 (0.89)	0 (0)	
kir7.2	123	(1.0)	359	(1.0)	0.323	102.40*	3.64^{NS}	0.131 (0.84)	2.64 (0.04)	
kita	140	(1.0)	987	(1.0)	0.255	555.91*	56.23*	0.067 (0.80)	3.89 (0.02)	32, 46, **62**, 64, **117**, 185, **193**, **195**, **394**, 745
kitla	141	(0.7)	268	(0.9)	0.907	158.57*	69.31*	0.027 (0.41)	5.68 (0.07)	40, 82, **83**, 109, **170**, **171**, 172, **192**, **193**
mitfa	140	(0.9)	401	(1.0)	0.137	116.26*	0^{NS}	0.044 (0.88)	0 (0)	
smtlb	142	(0.8)	230	(1.0)	0.268	51.38*	0^{NS}	0.103 (0.79)	0 (0)	
sox10	143	(1.0)	467	(0.5)	0.105	87.51*	0^{NS}	0.056 (0.93)	0 (0)	

Supplementary Table 7 (Cont.)

Genes involved in colour vision										
sws1	130	(0.9)	335	(1.0)	0.223	282.91*	11.01*	0.080 (0.82)	3.16 (0.02)	89, 107[e], 273
sws2b	135	(0.9)	352	(1.0)	0.469	220.31*	35.74*	0.055 (0.64)	3.92 (0.05)	115, **271**[e], 279[e], **310**, 340, 352
sws2a	134	(1.0)	352	(1.0)	0.358	290.14*	26.93*	0.069 (0.73)	2.97 (0.04)	53[e], 62, 105, 172, 238, 254, 265, 288
rh2b	136	(0.9)	217	(0.6)	0.440	126.28*	21.25*	0.126 (0.74)	3.19 (0.05)	**107,** 108, 127, 174
rh2aβ	131	(1.0)	297	(0.8)	0.360	745.12*	132.49*	0.040 (0.81)	3.80 (0.05)	**2**, **4**, 35, **107**, 157, **173**, 295, **312**, **339**, **343**
rh2aα	134	(0.9)	297	(0.8)	0.317	579.34*	94.85*	0.028 (0.84)	3.63 (0.05)	2, **4**, **32**, **35**, 43, 47, **107**, **157**, 159, **166**, 285, 292, 339, **343**
lws	134	(0.9)	357	(1.0)	0.376	655.42*	108.17*	0.036 (0.83)	3.40 (0.07)	**23**, **26**, 52, **62**, **63**, **76**, 168, **175**, **177**[e], **179**, 182, 185, 222, 226, **230**, **241**, 261, 272, **275**[e]
Genes involved in bone and tooth development										
col6a1	147	(0.9)	193	(0.2)	0.321	262.92*	28.91*	0.075 (0.73)	3.33 (0.05)	**20**, 24, 76, 116, 118, 189
c-fos	142	(1.0)	378	(1.0)	0.283	143.63*	8.21*	0.081 (0.84)	1.58 (0.16)	119
creb1	142	(0.8)	318	(1.0)	0.068	55.54*	0.42^{NS}	0.017 (0.92)	0 (0)	
barx1	139	(0.9)	244	(1.0)	0.082	44.44*	0^{NS}	0.042 (0.94)	0 (0)	
pitx2	141	(0.2)	68	(0.3)	0.014	0^{NS}	0^{NS}	0.014 (1)	0 (0)	
runx2b	147	(0.7)	304	(0.7)	0.018	26.64*	0^{NS}	0.010 (0.99)	1.02 (0.005)	
shh	142	(0.9)	415	(1.0)	0.149	103.95^{NS}	0^{NS}	0.055 (0.86)	0 (0)	
bmp2	147	(0.3)	118	(0.3)	0.089	4.81^{NS}	0^{NS}	0.089 (1)	0 (0)	
dlx2	141	(0.6)	276	(1.0)	0.168	8.09^{NS}	0^{NS}	0.168 (1)	0 (0)	
bmp4	146	(0.4)	118	(0.3)	0.095	10.24*	0.08^{NS}	0.024 (0.92)	0 (0)	
sp7	136	(0.9)	461	(1.0)	0.073	54.31*	0^{NS}	0.025 (0.95)	0 (0)	

[a] **s**: number of sequences; $\mathbf{p_{US}}$: proportion of unique sequences; **ls**: length of sequences; $\mathbf{p_{CDS}}$: proportion of the CDS captured.

[c] p_0: proportion of sites assigned to the class with ω_0; p_2: proportion of sites assigned to the class with ω_1; $\omega_1 = 1$; $p_1 = 1 - p_0 - p_2$

[d] Only sites with a posterior probability higher than 85% are reported. If the posterior probability of a site belonging to the positively selected class (ω_2) is $0.90>P>0.95$ the site number is underlined and if it is $\boldsymbol{P<0.95}$ it is in bold. Sites are numbered following bovine rhodopsin.

[e] Sites directed into the chromophore binding pocket.

Supplementary Table 8. Analysis of molecular divergence in bone and tooth (jaw) development genes showing evidences of positive selection. For each gene, different models are fitted to the data, which partition taxa by phylogeny (CmC-Tanganyika, CmC-Tanganyika-Haplochromini, CmC-Haplochromini) and diet, or no partition (null model M2a-rel[38]) (Fig. 4c). The significance (*) of each model is tested against the null model by LRT after Benjamini-Hochberg correction and the relative fit among different models compared by AIC. Values of the coefficient of selection are shown for the two assumed taxa partitions (background/ foreground) indicating their phenotype. Analyses performed with PAML clade C models, which assume three site classes: class 0 (negatively selected), class 1 (neutral sites; not shown) and class 2 (variable). Significance levels were corrected using the Benjamini-Hochberg correction for multiple testing, applied according to the numer genes for which each each hypothesis (i.e. clade C model) is tested.

				LRT	Site class 0		Site class 2				
Opsin gene	Model tested	*ln*L	∂AIC	2Δ*ln*L	ω	*p*	Background ω		Foreground ω		*p*
c-fos	CmC-diet	-4301.787	0	9.094^{NS}	0.053	(0.790)	benthos:	3.448	nekton:	0.753	(0.070)
							aufwuchs:	1.047	plankton:	0	
							omnivores:	0.342			
	M2a-rel	-4306.334	1.094		0.065	(0.811)		2.158			(0.075)
	CmC-Tanganyika	-4305.767	1.960	1.134^{NS}	0.065	(0.811)		1.684	LT:	2.507	(0.080)
	CmC-Haplochromini	-4306.013	2.453	0.642^{NS}	0.065	(0.810)		2.171	HC:	2.127	(0.075)
	CmC-Tanganyika-Haplochromini	-4305.338	3.101	1.993^{NS}	0.062	(0.803)		1.726	LT(-HC):	2.917	(0.065)
									HC:	2.087	
col6a1	CmC-Tanganyika-Haplochromini	-3880.245	0	28.378*	0.066	(0.745)		0.410	LT(-HC):	6.795	(0.052)
									HC:	0	
	CmC-diet	-3887.553	18.615	13.763*	0.068	(0.728)	benthos:	5.425	nekton:	0	(0.054)
							aufwuchs:	1.112	plankton:	3.653	
							omnivores:	2.410			
	CmC-Haplochromini	-3891.088	19.685	6.693^{NS}	0.070	(0.728)		3.584	HC:	0.345	(0.063)
	CmC-Tanganyika	-3893.351	24.211	2.168^{NS}	0.068	(0.728)		2.697	LT:	4.531	(0.052)
	M2a-rel	-3894.434	24.378		0.068	(0.727)		3.429			(0.055)

Abbreviations refer to: Lake Tanganyika flock (LT), Haplochromini (HC), Lake Tanganyika flock excluding Haplochromini (LT-(HC)).

Supplementary Table 9. Analysis of molecular divergence in coloration genes showing evidences of positive selection. For each gene, different models are fitted to the data, which partition taxa by phylogeny (Cmc-Tanganyika, CmC-Tanganyika-Haplochromini, CmC-Haplochromini), colour polymorphism, diet, or no partition (null model M2a-rel[38]) (Fig. 4c). The significance (*) of each model is tested against the null model by LRT after Benjamini-Hochberg correction and the relative fit of different models compared by AIC. Values of the coefficient of selection are shown for the two assumed taxa partitions (background/ foreground) indicating their phenotype. Analyses performed with PAML clade C models, which assume three site classes: class 0 (negatively selected), class 1 (neutral sites; not shown) and class 2 (variable).

				LRT	Site class 0		Site class 2				
Gene	Model tested	*ln*L	∂ AIC	2Δ*ln*L	ω	*P*		Background ω		Foreground ω	*p*
dlc	CmC-Haplochromini	-14594.488	0	142.122*	0.080	(0.805)		0.658	HC:	18.527	(0.035)
	CmC-Tanganyika-Haplochromini	-14593.584	0.192	143.929*	0.080	(0.804)		0.595	LT(-HC):	0.873	(0.034)
									HC:	18.667	
	CmC-Tanganyika	-14642.301	95.626	46.495*	0.083	(0.797)		2.108	LT:	5.933	(0.026)
	M2a-rel	-14665.549	140.122	–	0.228	(0.326)	–	0.022	–	–	(0.532)
	CmC-dimorphism	-14665.377	141.778	0.343^{NS}	0.230	(0.322)	m.c.:	0.017	d.c.:	0.024	(0.535)
csf1ra	CmC-Tanganyika-Haplochromini	-14806.609	0	30.362*	0.076	(0.787)		0.808	LT(-HC):	0.077	(0.047)
									HC:	1.089	
	CmC-Tanganyika	-14815.479	16.122	12.240*	0.070	(0.750)		0.800	LT:	0.080	(0.110)
	M2a-rel	-14821.599	26.362		0.094	(0.834)		0.768			(0.091)
	CmC-dimorphism	-14821.558	28.279	0.083^{NS}	0.073	(0.802)	m.c.:	0.691	d.c.:	0.793	(0.091)
	CmC-Haplochromini	-14821.584	28.332	0.029^{NS}	0.073	(0.802)		4.483	HC:	1.072	(0.091)
kita	Cmc-Tanganyika-Haplochromini	-14904.432	0.00	12.611*	0.067	(0.800)		2.834	LT(-HC):	6.492	(0.018)
									HC:	3.796	
	M2a-Tanganyika	-14905.785	0.706	9.906*	0.067	(0.800)		2.826	LT:	5.716	(0.018)
	M2a-el	-14910.738	8.611		0.068	(0.800)		3.684			(0.021)
	CmC-dimorphism	-14910.255	9.645	0.966^{NS}	0.068	(0.800)	m.c.:	3.828	d.c.:	2.721	(0.021)
	CmC-Haplochromini	-14910.638	10.412	0.200^{NS}	0.068	(0.800)		3.698	HC:	3.522	(0.021)

Supplementary Table 9 (Cont.)

kitla	CmC- Haplochromini	-3345.137	0	3.106^{NS}	0.047	(0453)		5.965	HC:	9.407	(0.059)
	CmC- Tanganyika	-3345.622	0.971	2.136^{NS}	0.048	(0.452)		6.141	LT:	6.232	(0.062)
	CmC-dimorphism	-3345.623	0.972	2.135^{NS}	0.048	(0.452)	m.c.:	6.168	d.c.:	6.109	(0.063)
	M2a-rel	-3346.690	1.106		0.048	(0.452)		6.159			(0.062)
	CmC-Tanganyika-Haplochromini	-3346.090	3.907	1.200^{NS}	0.048	(0.453)		6.131	LT(-HC):	5.279	(0.061)
									HC:	9.053	
hag	CmC-Haplochromini	-6967.975	0	42.354*	0.056	(0.833)		0.103	HC:	10.945	(0.017)
	CmC-Tanganyika-Haplochromini	-6967.004	0.058	44.297*	0.055	(0.832)		0.157	LT(-HC):	0	(0.018)
									HC:	10.474	
	CmC- Tanganyika	-6982.141	28.332	14.023*	0.060	(0.841)		0.547	LT:	2.989	(0.030)
	CmC-dimorphism	-6984.866	33.782	8.572*	0	(0.525)	m.c.:	0.129	d.c.:	0.216	(0.351)
	M2a-rel	-6989.153	40.354	–	0	(0.530)	–	0.206		–	(0.350)

Abbreviations refer to: Lake Tanganyika flock (LT), Haplochromini (HC), Lake Tanganyika flock excluding Haplochromini (LT-(HC)), sexually monomorphic in coloration (m.c.), sexually dimorphic in coloration (d.c.).

Supplementary Table 10. Analysis of molecular divergence in cone opsin genes. For each gene, different models are fitted to the data, which partition taxa by phylogeny (CmC-Tanganyika, CmC-Tanganyika-Haplochromini, CmC-Haplochromini) or no partition (null model M2a-rel[38]) (Fig. 4c). The significance (*) of each model is tested against the null model by LRT after Benjamini-Hochberg correction and the relative fit of different models compared by AIC. Values of the coefficient of selection are shown for the two assumed taxa partitions (background/foreground) indicating their phenotype. Analyses performed with PAML clade C models, which assume three site classes: class 0 (negatively selected), class 1 (neutral sites; not shown) and class 2 (variable).

				LRT	Site class 0		Site class 2			
Gene	Model tested	*ln*L	∂AIC	2Δ*ln*L	ω	*P*	Background ω		Foreground ω	*p*
sws1	CmC-Tanganyika-Haplochromini	-6114.840	0	101.667*	0.059	(0.780)	0.351	LT(-HC):	4.648	(0.160)
								HC:	0.824	
	CmC-Tanganyika	-6125.148	18.617	81.005*	0.058	(0.776)	0.967	LT:	3.274	(0.175)
	M2a-rel	-6165.673	97.667		0.073	(0.802)	4.106			(0.019)
	CmC-Haplochromini	-6164.853	98.026	1.641^{NS}	0.073	(0.802)	3.656	HC:	6.085	(0.019)
sws2b	CmC-Tanganyika	-4908.315	0	40.059*	0.073	(0.803)	1.545	LT:	8.332	(0.051)
	CmC-Tanganyika-Haplochromini	-4908.810	2.756	39.303*	0.073	(0.802)	1.507	LT(-HC):	9.487	(0.047)
								HC:	5.894	
	M2a-rel	-4928.461	38.059		0.074	(0.817)	5.039			(0.047)
	CmC-Haplochromini	-4928.461	41.395	0^{NS}	0.073	(0.802)	4.483	HC:	7.030	(0.054)
sws2a	CmC-Tanganyika	-5747.384	0	58.038*	0.068	(0.742)	0.971	LT:	6.426	(0.066)
	CmC-Tanganyika-Haplochromini	-5747.306	1.844	58.193*	0.068	(0.742)	0.973	LT(-HC):	6.574	(0.066)
								HC:	5.655	
	CmC- Haplochromini	-5775.373	55.978	2.059^{NS}	0.068	(0.741)	3.132	HC:	6.235	(0.058)
	M2a-rel	-5776.403	56.038		0.067	(0.739)	3.443			(0.054)
rh2b	CmC-Tanganyika-Haplochromini	-2982.055	0	43.544*	0.115	(0.777)	0.846	LT(-HC):	5.148	(0.165)
								HC:	1.381	
	CmC- Tanganyika	-2983.632	1.153	40.391*	0.115	(0.776)	0.847	LT:	4.847	(0.161)
	M2a-rel	-3003.827	39.544		0.105	(0.747)	3.137			(0.077)
	CmC- Haplochromini	-3003.789	41.467	0.077^{NS}	0.106	(0.748)	3.129	HC:	1.963	(0.081)

Abbreviations refer to: Lake Tanganyika flock (LT), Haplochromini (HC), Lake Tanganyika flock excluding Haplochromini (LT-(HC)).

Supplementary Table 10 (Cont.)

rh2aβ	CmC-Tanganyika	-5873.019	0	35.593*	0.027	(0.823)	1.121	LT:	5.243	(0.049)
	CmC-Tanganyika-Haplochromini	-5872.896	1.754	35.840*	0.027	(0.823)	1.241	LT(-HC):	5.407	(0.047)
								HC:	4.607	
	M2a-rel	-5890.816	33.593		0.027	(0.822)	4.042			(0.047)
	CmC-Haplochromini	-5890.747	33.456	0.137^{NS}	0.027	(0.822)	3.972	HC:	4.462	(0.048)
rh2aα	CmC-Tanganyika	-5107.105	0	11. 546*	0.021	(0.838)	2.006	LT:	4.732	(0.049)
	CmC-Tanganyika-Haplochromini	-5110.194	8.984	$4.\ 562^{NS}$	0.021	(0.838)	1.989	LT(-HC):	5.062	(0.048)
								HC:	3.782	
	M2a-rel	-5112.878	9.546		0.021	(0.838)	3.842			(0.052)
	CmC-Haplochromini	-5113.609	13.008	0^{NS}	0.021	(0.838)	3.846	HC:	3.795	(0.052)
lws	CmC-Tanganyika-Haplochromini	-5658.840	0	12.605*	0.025	(0.804)	4.498	LT(-HC):	2.913	(0.065)
								HC:	6.725	
	CmC- Haplochromini	-5661.373	5.978	5.587^{NS}	0.025	(0.804)	3.914	HC:	6.898	(0.062)
	CmC-Tanganyika	-5662.148	5.617	5.334*	0.024	(0.804)	4.774	LT:	3.771	(0.059)
	M2a-rel	-5664.403	8.038		0.025	(0.804)	4.270			(0.060)

Abbreviations refer to: Lake Tanganyika flock (LT), Haplochromini (HC), Lake Tanganyika flock excluding Haplochromini (LT-(HC)).

Supplementary Table 11. Analysis of molecular divergence in cone opsins. For each gene, five different models are fitted to the data that partition taxa by habitat (deep/shallow water; depth), breeding mode (mouth/ substrate brooding; breeding), sexual colour dimorphism (present/ absent; dimorphism), diet (benthos/ plankton/ nekton/ aufwuchs/ generalist) or no partition (null model M2a[38]). The significance (*) of each model is tested against the null model by LRT after Benjamini-Hochberg correction and the fit of different models compared by AIC. Values of the coefficient of selection are shown for the two assumed taxa partitions (background/foreground) indicating their phenotype. Analyses performed with PAML clade C models, which assume three site classes: class 0 (negatively selected), class 1 (neutral sites; not shown) and class 2 (variable).

				LRT	Site class 0		Site class 2				
Opsin gene	Model tested	*ln*L	∂AIC	2Δ*ln*L	ω	*p*	Background ω		Foreground ω		*p*
sws1	CmC-diet	-3469.485	0	24.550*	0	(0.643)	benthos:	0.784	nekton:	7.422	(0.064)
							aufwuchs:	0.641	plankton:	12.701	
	CmC-depth	-3475.728	8.485	12.065*	0	(0.644)	sw.d.:	5.967	dw.d	0	(0.077)
	CmC-breeding mode	-3478.628	14.286	6.264*	0	(0.644)	s.b.:	8.236	m.b.:	4.015	(0.070)
	CmC-dimorphism	-3479.094	15.218	5.333^{NS}	0	(0.644)	m.c.:	6.712	d.c.:	3.975	(0.069)
	M2a-rel	-3481.760	18.550		0	(0.644)		5.661			(0.072)
sws2b	CmC-diet	-3484.315	0	27.732*	0.009	(0.569)	benthos:	13.379	nekton:	0	(0.043)
							aufwuchs:	0.641	plankton:	13.730	
	CmC-dimorphism	-3490.315	7.844	15.888*	0.003	(0.567)	m.c.:	14.111	d.c.:	1.245	(0.036)
	CmC-breeding mode	-3491.279	9.928	13.804*	0.004	(0.560)	s.b.:	15.032	m.b.:	5.401	(0.042)
	CmC-depth	-3493.504	14.379	9.352*	0.012	(0.565)	sw.d.:	7.806	dw.d.:	25.252	(0.048)
	M2a-rel	-3498.181	21.732		0.012	(0.568)		9.285			(0.048)
sws2a	CmC-breeding mode	-3551.259	0	12.338*	0.059	(0.735)	s.b.:	9.121	m.b.:	3.627	(0.057)
	CmC-diet	-3550.153	1.789	14.549*	0.055	(0.732)	benthos:	8.379	nekton:	4.830	(0.055)
							aufwuchs:	0.007	plankton:	1.285	
	CmC-dimorphism	-3553.355	4.192	8.147*	0.066	(0.742)	m.c.:	8.100	d.c.:	3.312	(0.061)
	M2a-rel	-3557.428	10.338		0.084	(0.767)		6.284			(0.067)
	CmC-depth	-3556.787	11.056	1.282^{NS}	0.082	(0.764)	sw.d.:	6.489	dw.d.:	5.001	(0.067)

Supplementary Table 11 (Cont.)

rh2b	CmC-diet	-1986.071	0	13.844*	0	(0.575)	benthos:	7.523	nekton:	5.972	(0.101)
							aufwuchs:	0.0001	plankton:	7.535	
	CmC-breeding mode	-1990.620	5.097	4.747^{NS}	0	(0.574)	s.b.:	8.873	m.b.:	3.654	(0.103)
	CmC-depth	-1991.034	5.925	3.919^{NS}	0	(0.579)	sw.d.:	5.359	dw.d.:	33.178	(0.110)
	M2a-rel	-1992.993	7.844		0	(0.582)		5.932			(0.117)
	CmC-dimorphism	-1992.964	9.786	0.058^{NS}	0	(0.582)	m.c.:	6.064	d.c.:	5.388	(0.177)
rh2aβ	M2a-rel	-4726.929	0		0.020	(0.813)		5.485			(0.048)
	CmC-dimorphism	-4726.614	1.370	0.630^{NS}	0.020	(0.813)	m.c.:	5.735	d.c.:	4.824	(0.048)
	CmC-breeding mode	-4726.823	1.787	0.213^{NS}	0.020	(0.813)	s.b.:	5.711	m.b.:	5.218	(0.048)
	CmC-depth	-4726.837	1.815	0.185^{NS}	0.020	(0.813)	sw.d.:	5.555	dw.d.:	4.834	(0.048)
	CmC-diet	-4726.427	4.996	1.004^{NS}	0.020	(0.813)	benthos:	5.676	nekton:	5.334	(0.048)
							aufwuchs:	5.764	plankton:	3.960	
rh2aα	CmC-diet	-4062.860	0	9.117*	0.008	(0.839)	benthos:	5.523	nekton:	1.669	(0.038)
							aufwuchs:	7.407	plankton:	4.339	
	M2a-rel	-4067.418	3.117		0.009	(0.830)		4.681			(0.045)
	CmC-depth	-4066.571	3.423	1.694^{NS}	0.008	(0.829)	sw.d.:	4.804	dw.d.:	6.445	(0.041)
	CmC-breeding mode	-4066.769	3.819	1.298^{NS}	0.009	(0.831)	s.b.:	3.892	m.b.:	5.375	(0.052)
	CmC-dimorphism	-4067.130	4.541	0.576^{NS}	0.008	(0.830)	m.c.:	5.089	d.c.:	4.303	(0.041)
lws	CmC-depth	-3395.472	0	5.179^{NS}	0.031	(0.820)	sw.d.:	5.631	dw.d.:	1.655	(0.064)
	CmC-diet	-3394.904	2.864	6.316^{NS}	0.034	(0.826)	benthos:	5.906	nekton:	4.306	(0.067)
							aufwuchs:	1.653	plankton:	2.948	
	CmC-dimorphism	-3396.920	2.895	2.285^{NS}	0.043	(0.842)	m.c.:	4.289	d.c.:	6.191	(0.074)
	M2a-rel	-3398.062	3.179		0.036	(0.830)		5.027			(0.068)
	CmC-breeding mode	-3397.723	4.501	0.678^{NS}	0.037	(0.831)	s.b.:	4.521	m.b.:	5.496	(0.069)

Abbreviations refer to: swallow water dwellers (sw.d.), deep water dwellers (dw.d.), substrate-brooders (s.b.), moothbrooders (m.b.), sexually monomorphic in coloration (m.c.), sexually dimorphic in coloration (d.c.).

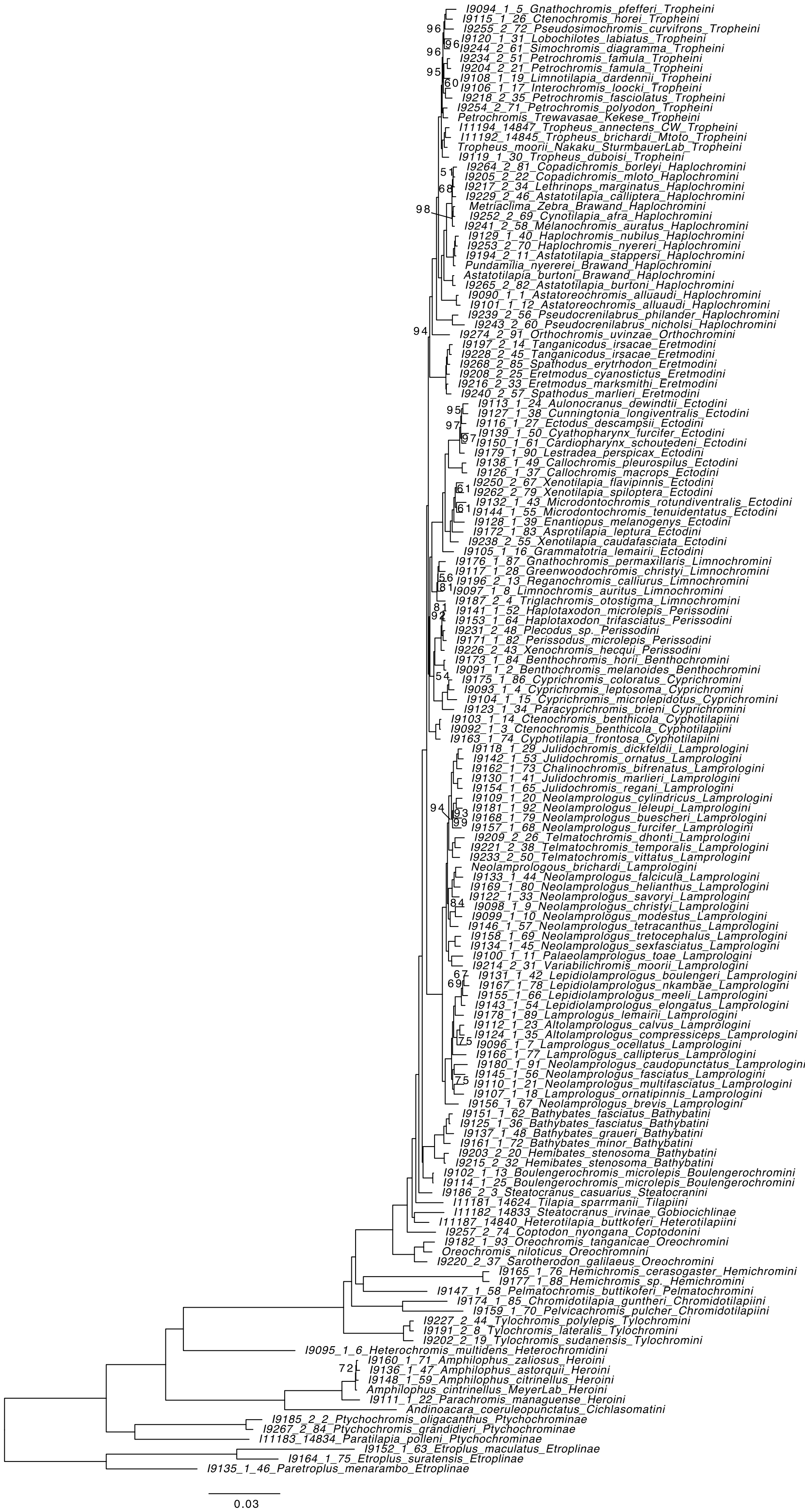

Supplementary Figure 1. Concatenated maximum likelihood phylogram (RAxML). All nodes received full (100%) support from non-parametric boostrapping, unless otherwise indicated. Scale bar is in expected substitutions site^{-1}.

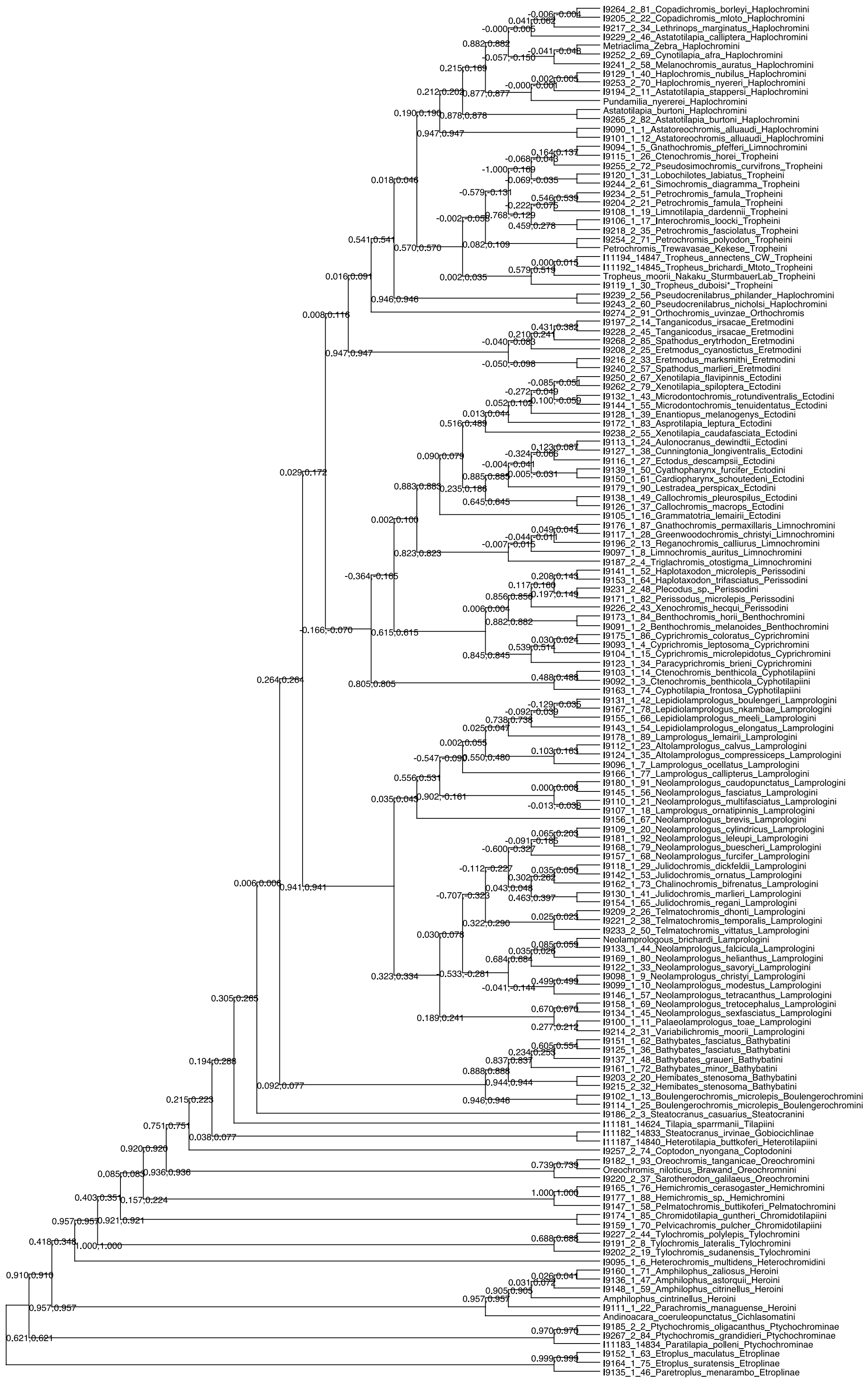

Supplementary Figure 2. Internode certainty values plotted onto the maximum likelihood tree (Supplementary Fig. 1). Number at nodes correspond respectively to internode certainty (IC) and internode certainty-all (ICA) values after probabilistic correction for partial locus trees

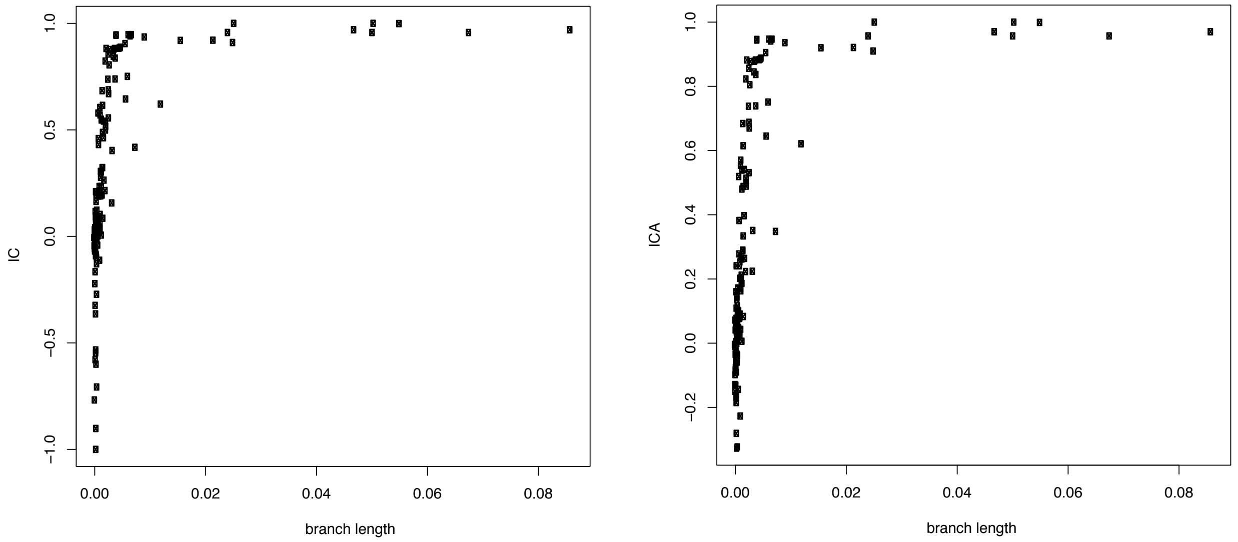

Supplementary Figure 3. Internode certainty (IC) and internode certainty-all (ICA) values plotted against branch lengths derived from the concatenated maximum likelihood phylogram. Low IC and ICA values derive mostly from short branches, suggesting the presence of incomplete lineage sorting.

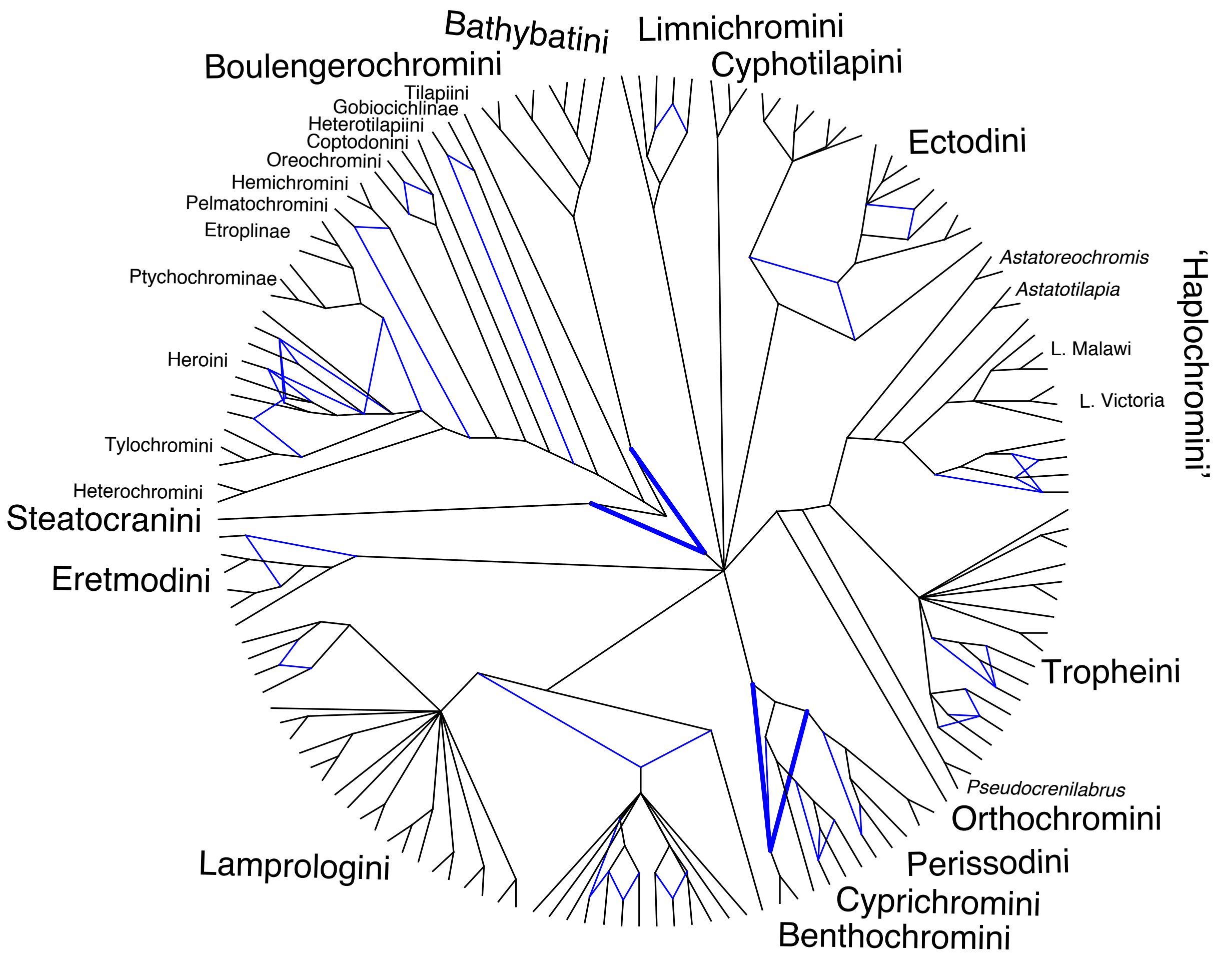

Supplementary Figure 4. Galled network. Reticulations supported by >20% locus trees shown in blue. Blue thick lines denote intertribal reticulations in the Lake Tanganyika species flock.

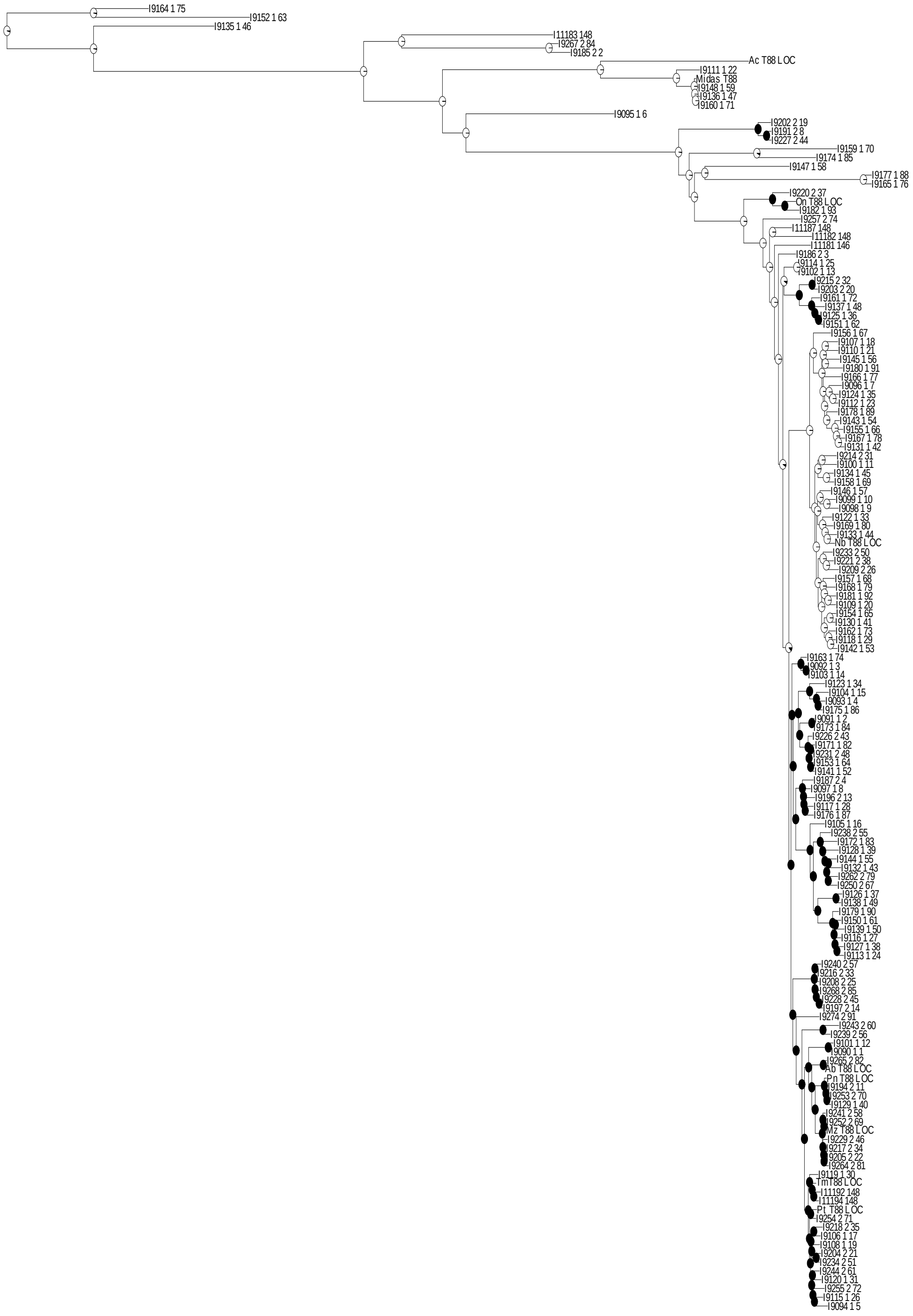

Supplementary Figure 5. Ancestral character state reconstruction for breeding mode. Circles at nodes represent posterior probablity for the two character states: substrate (white) and mouth-brooders (black).

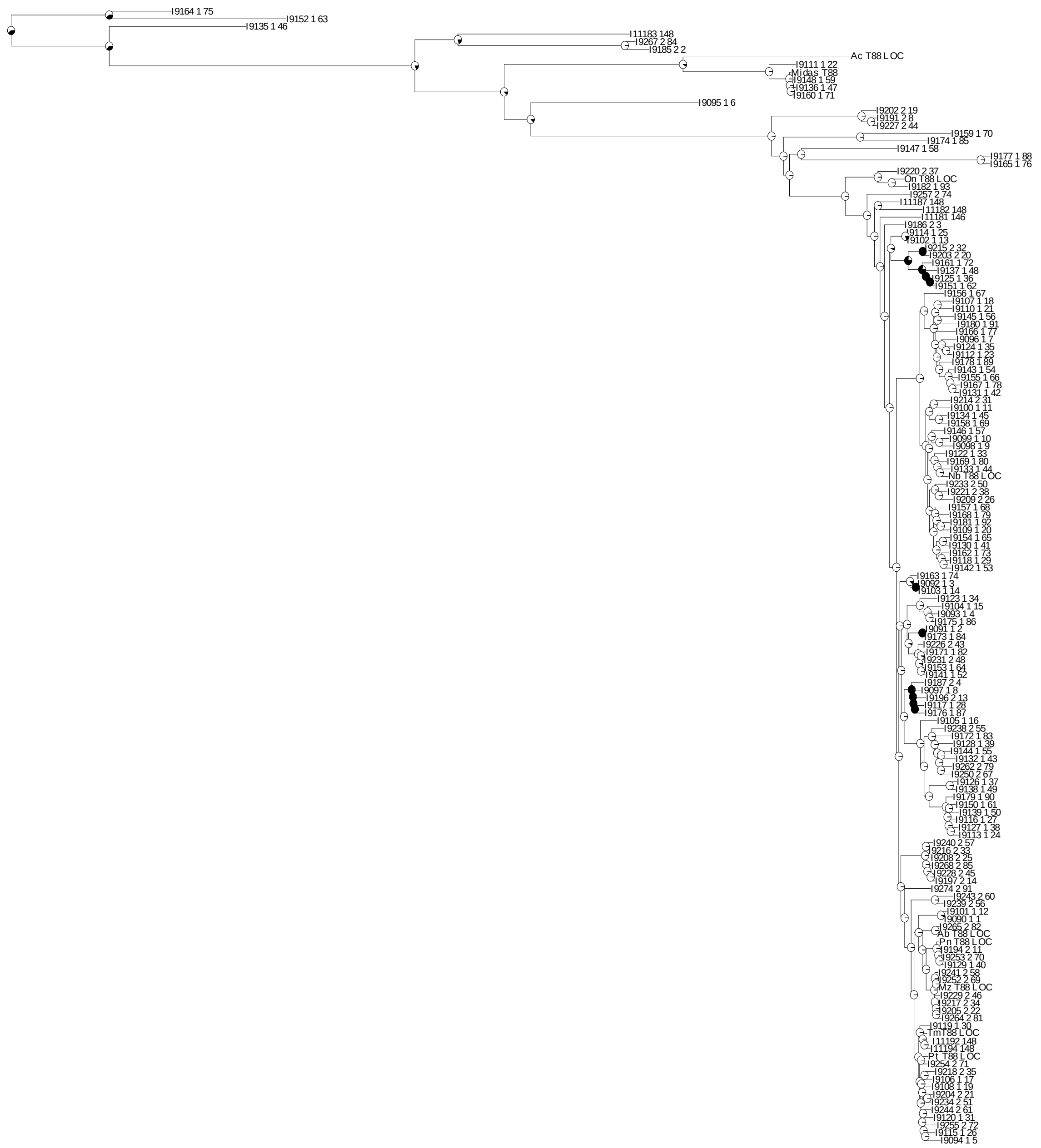

Supplementary Figure 6. Ancestral character state reconstruction for habitat. Circles at nodes represent posterior probablity for the two character states: shallow-water (white) and deep-water (black).

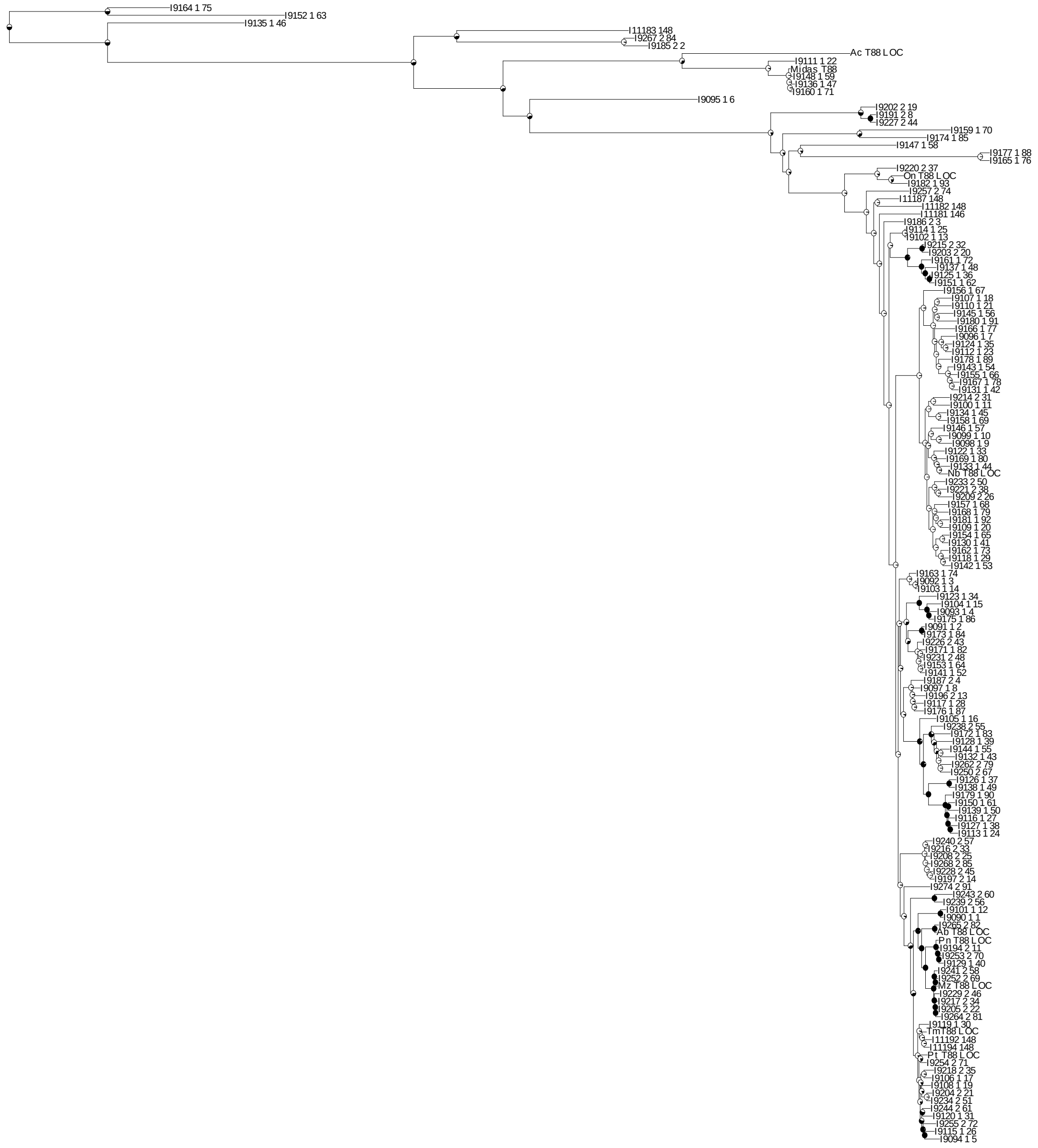

Supplementary Figure 7. Ancestral character state reconstruction for the presence of sex-related colour polymorphism. Circles at nodes represent posterior probablity for the two character state: absence (white) and presence (black).

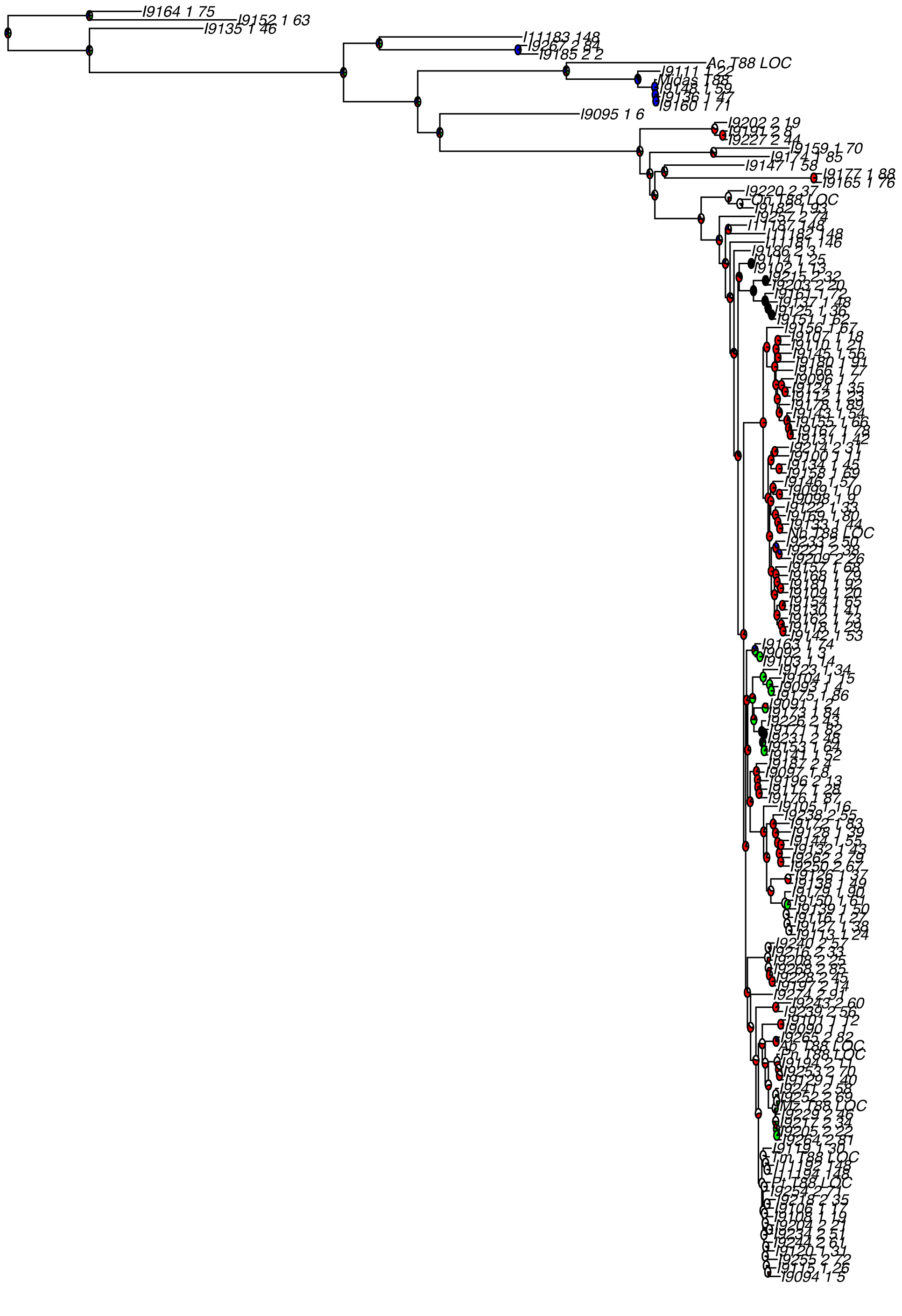

Supplementary Figure 8. Ancestral character state reconstruction for diet. Circles at nodes represent posterior probability for the five character states: generalist (blue), Aufwuchs (white), zoobenthos (red), nekton (black), and plankton (green).

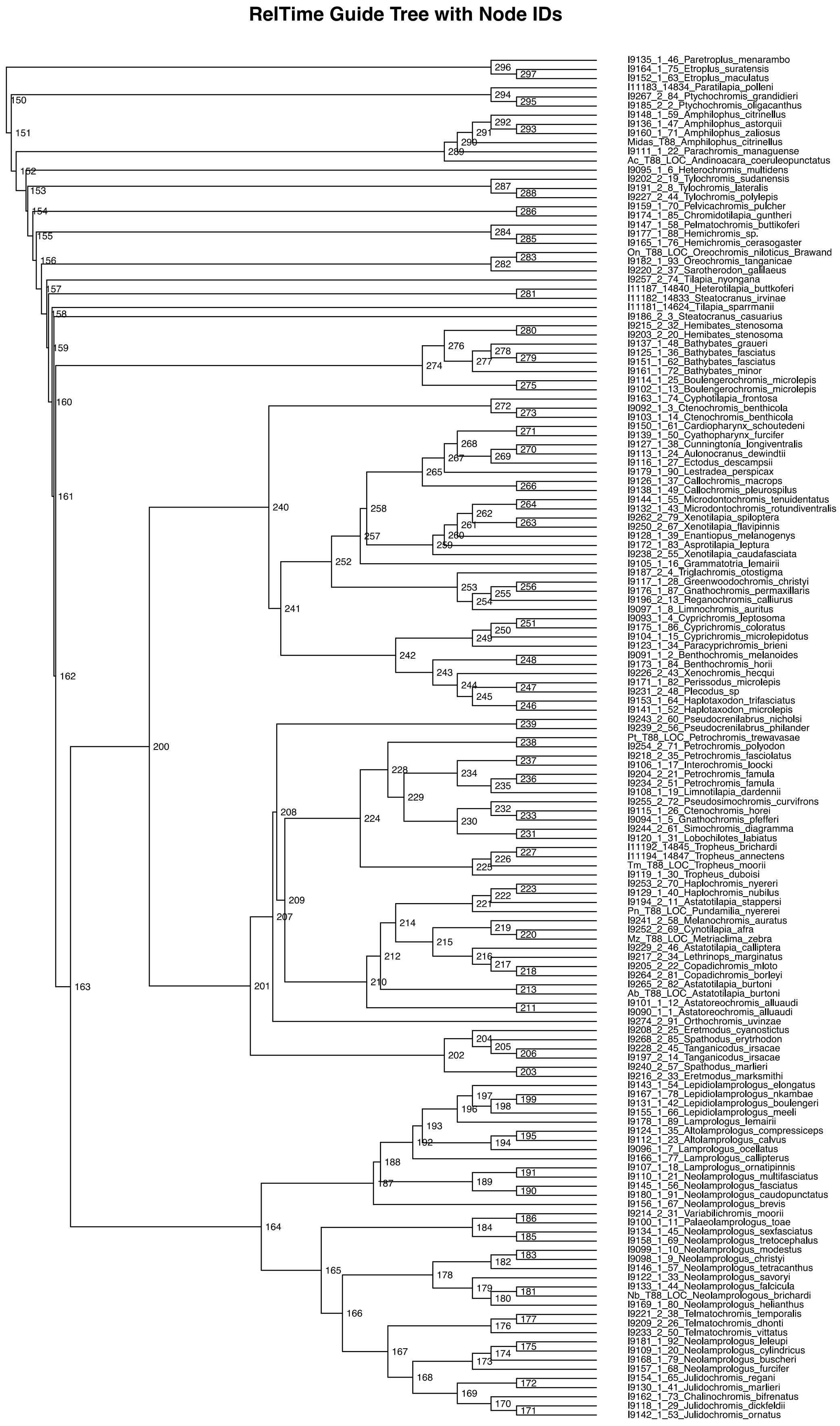

Supplementary Figure 9. Tree showing node IDs corresponding to estimated divergence times in Supplementary Data 1.

Supplementary References

1. Vences, M., Freyhof, J., Sonnenberg, R., Kosuch, J. & Veith, M. Reconciling fossils and molecules: Cenozoic divergence of cichlid fishes and the biogeography of Madagascar. *J. Biogeogr.* **28,** 1091–1099 (2001).
2. Genner, M. J. *et al.* Age of cichlids: new dates for ancient lake fish radiations. *Mol. Biol. Evol.* **24,** 1269–1282 (2007).
3. Del Papa, C. Estratigrafía y paleoambientes de la Formación Lumbrera, Grupo Salta, Noroeste Argentino. *Rev. Asoc. Geol. Argentina* **61,** 313–327 (2006).
4. Malabarba, M. & Malabarba, L. A new cichlid *Tremembichthys garciae* (Actinopterygii, Perciformes) from the Eocene-Oligocene of Eastern Brazil. *Rev. Bras. Paleontol.* **11,** 59–68 (2008).
5. Perez, P., Malabarba, M. & Del Papa, C. A new genus and species of Heroini (Perciformes: Cichlidae) from the early Eocene of southern South America. *Neotrop. Ichthy.* **8,** 631–642 (2010).
6. Murray, A. M. The oldest fossil cichlids (Teleostei: Perciformes): indication of a 45 million-year-old species flock. *Proceedings. Biol. Sci.* **268,** 679–684 (2001).
7. Murray, A. Eocene cichlid fishes from Tanzania, East Africa. *J. Vert. Paleontol.* **20,** 651–664 (2000).
8. Harrison, T. *et al.* Paleontological investigations at the Eocene locality of Mahenge in north-central Tanzania, East Africa. in *Eocene biodiversity: Unusual occurrences and rarely sampled habitats* (ed. Gunell, G. F.) 39–74 (Plenum Publishers, 2001).
9. Van Couvering, J. *Fossil cichlid fish of Africa. Special Papers in Paleontology* **29,** (The Paleontological Association, 1982).
10. Murray, A. & Stewart, K. A new species of tilapiine cichlid from the Pliocene, middle Awash, Ethiopia. *J. Vert. Paleontol.* **19,** 293–301 (1999).
11. Carnevale, G., Sorbini, C. & Landini, W. *Oreochromis lorenzoi*, a new species of tilapiine cichlid from the late Miocene of Central Italy. *J. Vert. Paleontol* **23,** 508–516 (2003).
12. Murray, A. Late Eocene and early Oligocene teleost and associated ichthyofauna of the Jebel Qatrani Formation, Fayum, Egypt. *Palaeontology* **47,** 711–724 (2004).
13. Altner, M., Schliewen, U. K., Penk, S. B. R. & Reichenbacher, B. †*Tugenchromis pickfordi*, gen. et sp. nov., from the upper Miocene—a stem-group cichlid of the 'East African Radiation'. *J. Vertebr. Paleontol.* **37,** e1297819 (2017).
14. Carleton, K. Cichlid fish visual systems: mechanisms of spectral tuning. *Integr. Zool.* **4,** 75–86 (2009).
15. Salzburger, W., Braasch, I. & Meyer, A. Adaptive sequence evolution in a color gene involved in the formation of the characteristic egg-dummies of male haplochromine cichlid fishes. *BMC Biol.* **5,** 51 (2007).
16. Hamada, H. *et al.* Involvement of Delta/Notch signaling in zebrafish adult pigment stripe patterning. *Development* **141,** 318–324 (2014).
17. Henning, F., Lee, H. J., Franchini, P. & Meyer, A. Genetic mapping of horizontal stripes in Lake Victoria cichlid fishes: Benefits and pitfalls of using RAD markers for dense linkage mapping. *Mol. Ecol.* **23,** 5224–5240 (2014).
18. Kawakami, K. *et al.* Proviral insertions in the zebrafish hagoromo gene, encoding an F-box/WD40-repeat protein, cause stripe pattern anomalies. *Curr. Biol.* **10,** 463–466 (2000).
19. Terai, Y., Morikawa, N., Kawakami, K. & Okada, N. The complexity of alternative splicing of hagoromo mRNAs is increased in an explosively speciated lineage in East African cichlids. *Pro. Natl. Acad. Sci. USA* **100,** 12798–12803 (2003).
20. Terai, Y., Morikawa, N., Kawakami, K. & Okada, N. Accelerated evolution of the surface amino acids in the WD-repeat domain encoded by the hagoromo gene in an explosively speciated lineage of East African cichlid fishes. *Mol. Biol. Evol.* **19,** 574–578 (2002).
21. Inaba, M., Yamanaka, H. & Kondo, S. Pigment pattern formation by contact-dependent depolarization. *Science* **335,** 677 (2012).
22. Singh, A. P., Schach, U. & Nusslein-Volhard, C. Proliferation, dispersal and patterned aggregation of iridophores in the skin prefigure striped colouration of zebrafish. *Nat. Cell Biol.* **16,** 607–614 (2014).
23. Watanabe, M., Hiraide, K. & Okada, N. Functional diversification of kir7.1 in cichlids accelerated

by gene duplication. *Gene* **399,** 46–52 (2007).
24. Parichy, D. M., Rawls, J. F., Pratt, S. J., Whitfield, T. T. & Johnson, S. L. Zebrafish sparse corresponds to an orthologue of c-kit and is required for the morphogenesis of a subpopulation of melanocytes, but is not essential for hematopoiesis or primordial germ cell development. *Development* **126,** 3425–3236 (1999).
25. Miller, C. T. *et al.* cis-Regulatory changes in Kit ligand expression and parallel evolution of pigmentation in sticklebacks and humans. *Cell* **131,** 1179–1189 (2007).
26. Tachibana, M. *et al.* Cloning of MITF, the human homolog of the mouse microphthalmia gene and assignment to chromosome 3p14.1-p12.3. *Hum. Mol. Genet.* **3,** 553–557 (1994).
27. Tassabehji, M., Newton, V. E. & Read, A. P. Waardenburg syndrome type 2 caused by mutations in the human microphthalmia (MITF) gene. *Nat. Genet.* **8,** 251–255 (1994).
28. McGill, G. G. *et al.* Bcl2 regulation by the melanocyte master regulator Mitf modulates lineage survival and melanoma cell viability. *Cell* **109,** 707–718 (2002).
29. Fukamachi, S., Sugimoto, M., Mitani, H. & Shima, A. Somatolactin selectively regulates proliferation and morphogenesis of neural-crest derived pigment cells in medaka. *Proc. Natl. Acad. Sci. USA* **101,** 10661–10666 (2004).
30. Fukamachi, S. *et al.* Dual control by a single gene of secondary sexual characters and mating preferences in medaka. *BMC Biol.* **7,** (2009).
31. Fukamachi, S., Yada, T., Meyer, A. & Kinoshita, M. Effects of constitutive expression of somatolactin alpha on skin pigmentation in medaka. *Gene* **442,** 81–87 (2009).
32. Dutton, K. A. *et al.* Zebrafish colourless encodes sox10 and specifies non-ectomesenchymal neural crest fates. *Development* **128,** 4113–4125 (2001).
33. Fraser, G. J. *et al.* An ancient gene network is co-opted for teeth on old and new jaws. *PLoS Biol.* **7,** e31 (2009).
34. Gunter, H. M. *et al.* Shaping development through mechanical strain: the transcriptional basis of diet-induced phenotypic plasticity in a cichlid fish. *Mol. Ecol.* **22,** 4516–4531 (2013).
35. Betancur, P., Bronner-Fraser, M. & Sauka-Spengler, T. Assembling neural crest regulatory circuits into a gene regulatory network. *Annu. Rev. Cell Dev. Biol.* **26,** 581–603 (2010).
36. Helms, J. A., Cordero, D. & Tapadia, M. D. New insights into craniofacial morphogenesis. *Development* **132,** 851–861 (2005).
37. Schneider, R. F., Li, Y., Meyer, A. & Gunter, H. M. Regulatory gene networks that shape the development of adaptive phenotypic plasticity in a cichlid fish. *Mol. Ecol.* **23,** 4511–4526 (2014).
38. Weadick, C. J. & Chang, B. S. W. An improved likelihood ratio test for detecting site-specific functional divergence among clades of protein-coding genes. *Mol. Biol. Evol.* **29,** 1297–1300 (2012).
