# Supplementary material for: Phylogenomics uncovers early hybridization and adaptive loci shaping the radiation of Lake Tanganyika cichlid fishes
Source: Nat Commun. 2018 Aug 8;9:3159. doi: 10.1038/s41467-018-05479-9 (PMC6082878; doi:10.1038/s41467-018-05479-9)
Supplement: Supplementary file 3 — Description of Additional Supplementary Files [file 41467_2018_5479_MOESM3_ESM.pdf]

Descriptions of Additional Supplementary Files

File Name: Supplementary Dataset 1
Description: Molecular dating results

File Name: Supplementary Dataset 2
Description: Patterson's D test results
